# A T-junction device allowing for two simultaneous orthogonal views: application to bubble formation and break-up

**DOI:** 10.1007/s10404-018-2101-1

**Published:** 2018-07-30

**Authors:** Davide Caprini, Giorgia Sinibaldi, Luca Marino, Carlo Massimo Casciola

**Affiliations:** 1 Dipartimento di Ingegneria Meccanica e Aerospaziale, Universitá di Roma La Sapienza, Via Eudossiana 18, 00184 Roma, Italy; 2 Center for Life Nano Science, Istituto Italiano di Tecnologia, Viale Regina Elena 291, 00163 Roma, Italy

**Keywords:** Micro-particle image velocimetry, Bubble dynamics

## Abstract

A novel design for the classical microfluidic device known as T-junction is proposed with the purpose of obtaining a simultaneous measurement of the in-plane velocity components in two orthogonal planes. A crucial feature of the proposed configuration is that all three velocity components are available along the intersection of the two planes. A dedicated optical set-up is developed to convey the two simultaneous views from the orthogonal planes into the sensor of a single camera, where a compound image is formed showing on either half one of the two views. A commercial micro-particle image velocimetry system is used to measure the velocity in the two planes. Feeding the T-junction with a liquid continuous phase and a dispersed gas phase, the velocity is measured by phase averaging along the bubble formation and break-up process showing the potentialities of the new design. The accuracy analysis shows that the error is dominated by a systematic component due to the thickness of the measurement slice. The error can be reduced by applying confocal microscopy to the present system with no further modifications so as to reduce the thickness of the measurement slab thereby reducing the error. Moreover, by sweeping the planes across the region of interest, a full three-dimensional reconstruction of the velocity field can be readily obtained. Finally, the simultaneous views offer the possibility to extract the principal curvatures of the bubble meniscus thereby providing access to the Laplace pressure.

## Introduction

In microfluidics, different methods are used to produce microbubbles, exploiting laminar flow conditions to allow for high reproducibility and high production rate at relatively low energy and mass transfer rates (Chen et al. 2014). Among the different devices conceived to manipulate fluids at the microscale (Whitesides and Stroock 2001), the T-junction is one of the most fundamental and widely used device. It consists of two orthogonally intersecting microchannels. The device is used to produce bubbles or droplets of a secondary phase into the main continuous one. Since its original development in the first few years of the present century, see, e.g., Thorsen et al. (2001), it has been repeatedly addressed by a number of investigators (Tice et al. 2003; Günther et al. 2004; Garstecki et al. 2006; Menech et al. 2008). In micro electro-mechanical systems (MEMS) and lab-on-chip (LOC), such geometry can be exploited to obtain two-phase flows (Zhao and Middelberg 2011), where two partially miscible or immiscible fluids interact in the microfluidic networks with well-defined and controlled conditions (Graaf et al. 2005; Garstecki et al. 2006; Fu and Ma 2015. The possible applications are countless. In the medical/biological field, LOCs featuring the T-junction configuration have been developed to deliver drugs at a precisely controlled rate, e.g., in Okushima et al. (2004) and Stride and Edirisinghe (2008). In micro- and nano-technology, T-shaped channels have been employed to produce regular-sized polymeric particles (Nisisako et al. 2004) typically used, e.g., in liquid chromatography (Ugelstad et al. 1983) or for flow measurements (PIV and microPIV) (Melling 1997). The generation of microbubbles is also relevant for fabricating porous biomaterials (Wang et al. 2011), for mixing enhancement in chemical processes (Günther et al. 2005; Kreutzer et al. 2005) and for different biomedical applications, e.g., to form liposomes (Swaay 2013).

Since two immiscible fluids are present, surface tension is dynamically important. On the other hand, given the scale of the order of hundreds micrometres and the velocities of tens of millimetres per second, inertial effects are usually negligible and viscous forces dominate over inertial forces. In these conditions, the Navier–Stokes equation can be linearised to describe a “creeping flow”. However, although the familiar convective non-linearity of macroscopic fluid mechanics is ineffective, instability may still set in due to competition between surface tension and viscous forces (Landau and Lifshits 1959; Taylor 1934). As a consequence, despite the simple geometry, the dynamics leading to bubble/droplet formation is far from being trivial. In particular, the complex bubble shape and the strong three-dimensional and time-dependent velocity and pressure distributions are rather difficult to measure directly when the characteristic size falls below the millimetric range. In simple cases, important information can be inferred from numerical simulations (Menech et al. 2008; Soh et al. 2016; Steijn et al. 2010; Amaya-Bower and Lee 2011).

However, a way to resort to experiments is clearly needed both for validation of the numerical models and for the investigation of more complex cases when, e.g., rheologically complex fluids are involved. The experimental analysis has been mainly focused on the global characterisation of the device addressing the influence of global parameters, such as characteristic length scale, flow rates and capillary number (Yamamoto and Ogata 2013; Wehking et al. 2014; Nunes et al. 2013; Fu et al. 2010). Attention has been given to unsteady phenomena, as in the case of bubble break-up (Garstecki et al. 2006; Fu et al. 2011; Fu and Ma 2015).

The velocity field can be obtained using micro-particle image velocimetry ($\mu$PIV), a non-invasive technique that allows to measure the velocity on planar sections (Steijn et al. 2007; Sinibaldi and Romano 2017). The accuracy is limited by the thickness of slab over which the field is implicitly averaged. A better resolution may be achieved by coupling $\mu$PIV with confocal microscopy, to reduce the thickness of the measurement volume, see Lima et al. (2006) and Oishi et al. (2009) for applications to the T-junction configuration. Still working with planar section, the third component of velocity can be acquired using stereo PIV (Lindken et al. 2006) at the prize of a considerably more complex system. By sweeping the measurement plane across the flow domain, a complete three-dimensional field can be reconstructed from the two-dimensional fields.

The purpose of this paper is to illustrate the concept of a novel set-up able to allow for the simultaneous measurement of the two-dimensional velocity field in two orthogonal planes in the T-junction. The fabrication technique used to build the proof-of-concept is described in some detail, but the reader should be aware that other alternative procedures and materials can be used. The process we adopted should then only be considered as an inexpensive and easy way to manufacture the microchip.

Concerning the velocity measurement, the advantage of the new configuration is that a standard $\mu$PIV is used, with a single camera. Along the intersection of the two orthogonal planes, all three velocity components are simultaneously acquired. In principle, by letting the intersection line span the measurement volume, the entire three-dimensional field can be reconstructed. After validation in a simple straight channel, the new approach is used in a T-junction to show how the flow field around the bubble and the bubble interface can be extracted during generation and break-up phases by phase averaging the combined planar acquisitions. The new set-up is based on the idea of looking at the field from two orthogonal planes. The two views are conveyed to the camera and each one is captured on one half of the sensor producing a compound image of the two sights. A standard PIV processing of the image allows to extract the velocity. The availability of the two orthogonal views may also be used to estimate the total curvature of the bubble/droplet, providing access to the Laplace pressure.

The paper is organised as follows. The experimental set-up is described in Sect. 2 where a detailed description of the new device is provided, including manufacturing, measurement technique and validation. The main results are presented in Sect. 3 where velocity field and bubble configuration are discussed. Finally, Sect. 4 is devoted to conclusions and possible perspectives. The three appendices complement the discussion with a detailed description of the fabrication procedure and a theoretical model of the measurement process exploited to assess accuracy and main sources of error.

## Experimental set-up

**Fig. 1:**
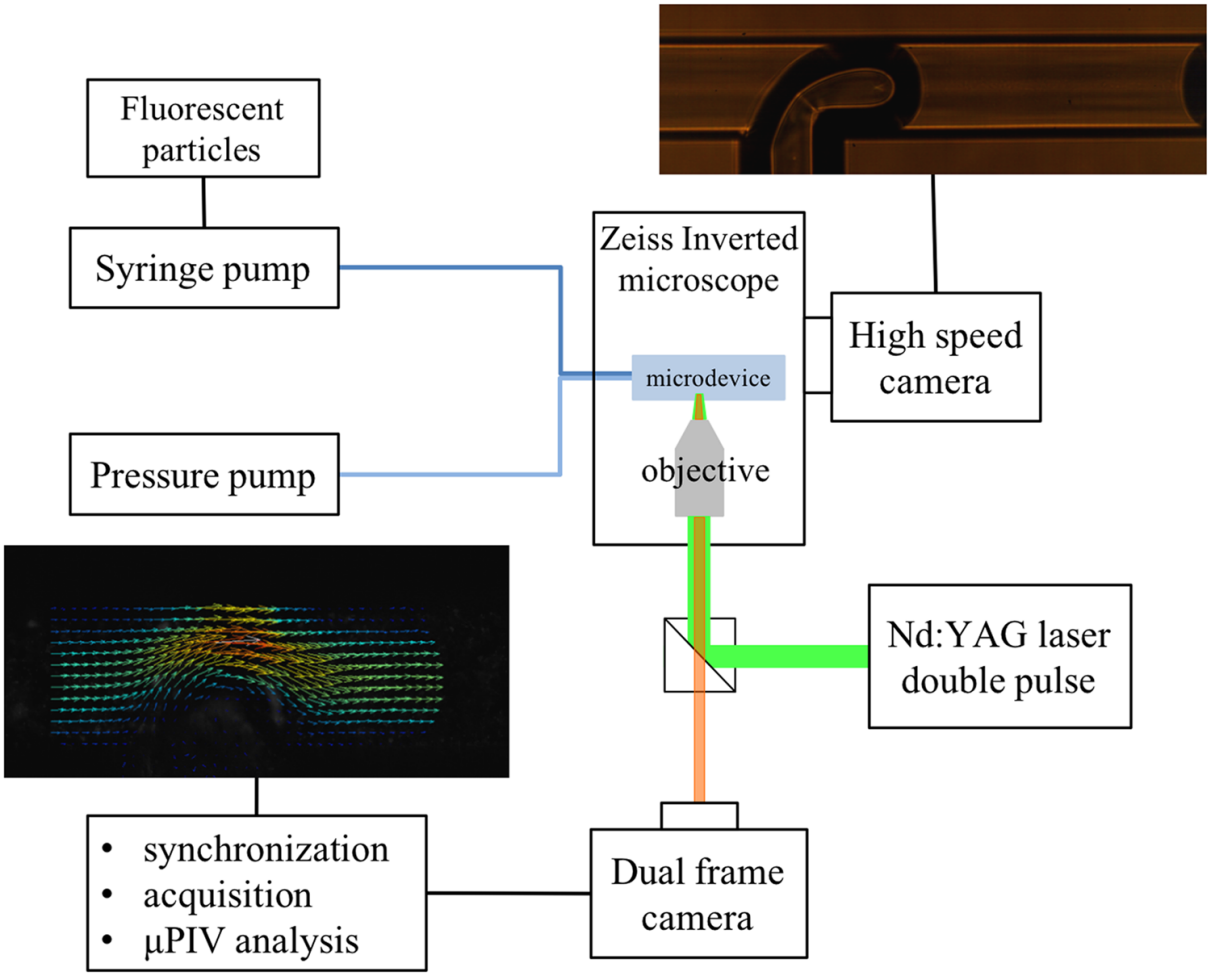
Sketch of the experimental set-up illustrating $\mu$PIV and high speed imaging. The fluidic circuit consists of a T-junction positioned under the objective of an inverted microscope. A syringe pump feeds the main channel with the liquid (continuous) phase (2-propanol), while a pressure pump is used for the secondary channel (dispersed phase)

The proposed device has been characterised by $\mu$PIV and high speed imaging, as schematised in Fig. 1. The latter is obtained by connecting a high speed camera (Photron mini UX100, $1280\times 1024$px CMOS sensor, 4000 fps at full frame and 800000 fps 1D) to an inverted microscope (Zeiss Observer Z1), provided with a 5$\times$ objective. For the $\mu$PIV system (LaVision), the same inverted microscope is combined with a Nd:YAG double pulsed laser (Litron NanoPIV) at 532 nm with a maximum pulse energy of 30 mJ and a pulse duration of 8 ns. The continuous phase is seeded with polystyrene, fluorescent particles with nominal diameter $D =4.47\, \upmu$m. The fluorescent light ($\lambda = 607$ nm) emitted by the microparticles is sent to a dual frame CCD camera (Imager SX-4) that captures pairs of images ($2360 \times 1776$ px) processed by the LaVision Davis software to provide the velocity field. The thickness of the volume where the velocity is measured, the depth of correlation of the $\mu$PIV system, depends mainly (Meinhart et al. 2000) on the numerical aperture of the objective, NA = 0.16, the magnification, M = 5$\times$, the light wavelength $\lambda$ and the particle diameter *D*. Under the present conditions, the correlation depth is $\ell _\mathrm{{c}} = 90 \, \upmu \mathrm{m}$. The interrogation window for $\mu$PIV analysis is $24 \times 24$ px with an overlap factor of $50 \, \%$ where $1 \, {\mathrm{px}}$ corresponds to $1.1 \, \upmu \mathrm{m}$. For the present case, the polystyrene fluorescent particles (microparticles GmbH) are provided as a suspension in water with mass ratio $r_\mathrm{{m}} = m_\mathrm{{p}}/m_\mathrm{{s}} = 2.5 \, \%$ where $m_\mathrm{{p}}$ and $m_\mathrm{{s}}$ are the mass of the particles and the mass of solution, respectively. Given the mass density of polystyrene, $\rho _\mathrm{{p}} = 1.050\, {\mathrm{g/cm}}^3$, $r_\mathrm{{m}}$ corresponds to the volume ratio $r_\mathrm{{v}} \simeq 2.5 \times 10^{-2}$. Before using the particles as $\mu$PIV tracers, the suspension is diluted in 2-propanol in the ratio 1:133. This corresponds to a volume fraction of polystyrene particles in the flowing solution of 2-propanol (plus water in traces) of $\phi \simeq 1.9 \times 10^{-4}$, resulting in a probability, occupancy fraction, $f_\mathrm{{o}} = 0.21$ of finding a particle in the interrogation volume. In other words, one out of (almost) five acquired images contains a velocity signal per interrogation volume. On the other hand, the probability of finding more than one particle per interrogation volume is negligible ($\sim 0.04$) thereby considerably simplifying the error analysis. It should be mentioned that such a low particle concentration also enhances particle visibility (Olsen and Adrian 2000), a parameter of general relevance in $\mu$PIV which is even more crucial in the present application where, as explained in the following section, two views with different optical paths need to be imaged.

### Device description and manufacturing

The microdevice consists of a T-junction with main microchannel for the liquid phase and secondary channel for the dispersed gas phase. The liquid phase is supplied by a syringe pump (PHD Ultra, Harvard Apparatus) with a mass flow rate in the range $1.56\, \mathrm{pl/min} - 216\, \mathrm{ml/min}$. The gas is fed by a pressure pump (Dolomite Mitos P-Pump) which keeps the control chamber pressure constant. The pump guarantees a steady flow over a wide pressure range (0–10 bar) with excellent response time and accuracy.

**Fig. 2:**
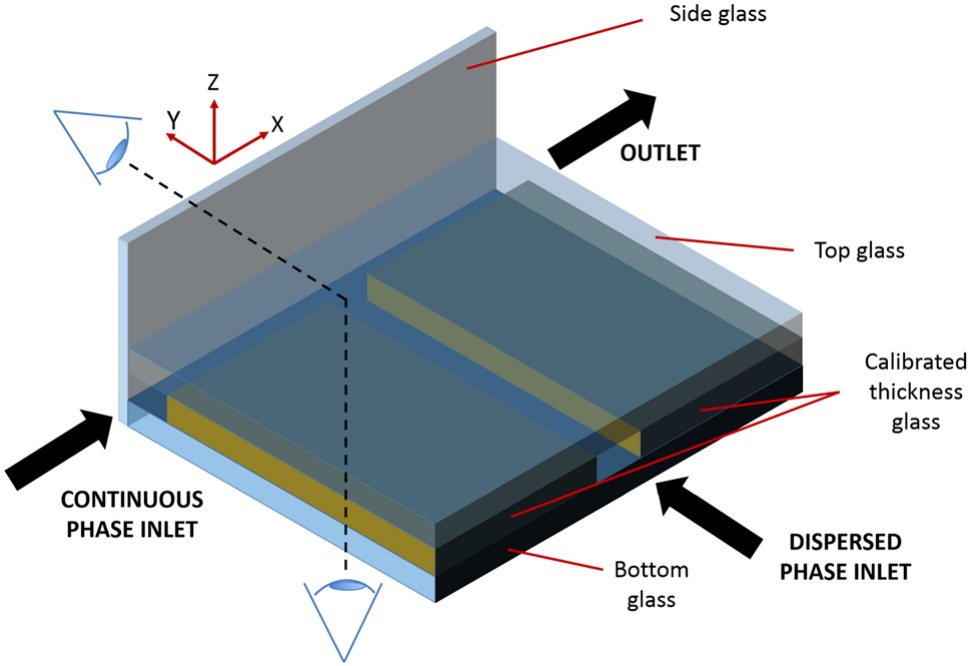
Three-dimensional schematic view of the novel chip. The two orthogonal views correspond to the *x*–*y* plane (direct view) and to the *z*–*x* plane (reflected view), respectively. (Color figure online)

**Fig. 3:**
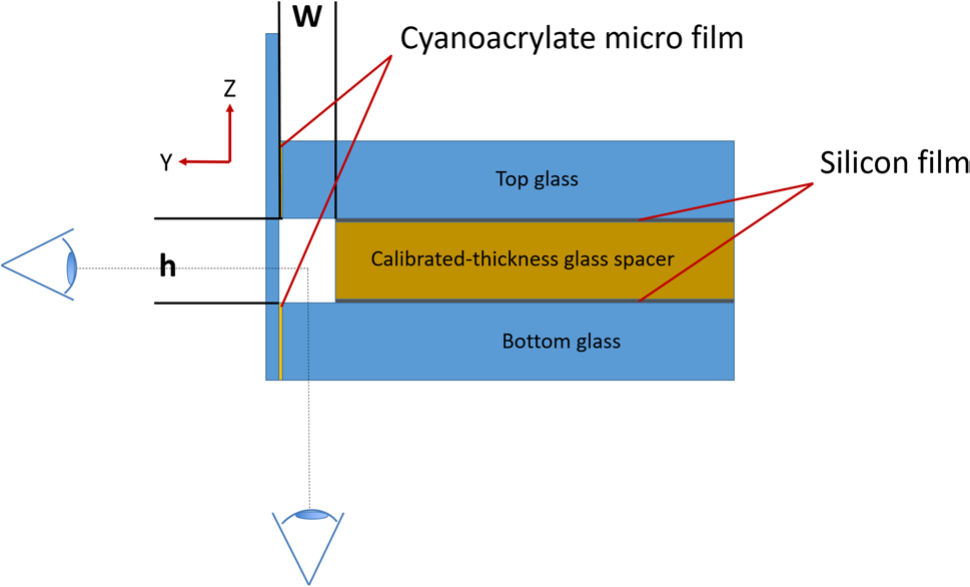
Sketch illustrating the chip cross-section to highlight the two orthogonal views and constructive details. (Color figure online)

We report here a brief description of the assembly procedure of the novel microdevice. The detailed fabrication process is reported in Appendix A. The novel chip is entirely built in glass, using calibrated slides to assemble the geometry sketched in Figs. 2 and 3. The main channel runs along one of the edges of the chip and offers a double optical access through the two orthogonal bottom and side slides joining at the bottom corner. The slides are glued together with a thin film of cyanoacrylate that can easily penetrate in the gap between the parts. The other elements are assembled using silicon glue, that allows for a precise positioning of calibrated spacers and top slide and prevents delamination due to the pressurised fluids. The adopted gluing procedure is an inexpensive and easy way to ensure proper bonding and perfect sealing in the investigated flow regimes. Alternative techniques, like thermal fusion bonding (Stjernström and Roeraade 1998; Jia et al. 2004), can be used if desired.

**Fig. 4:**
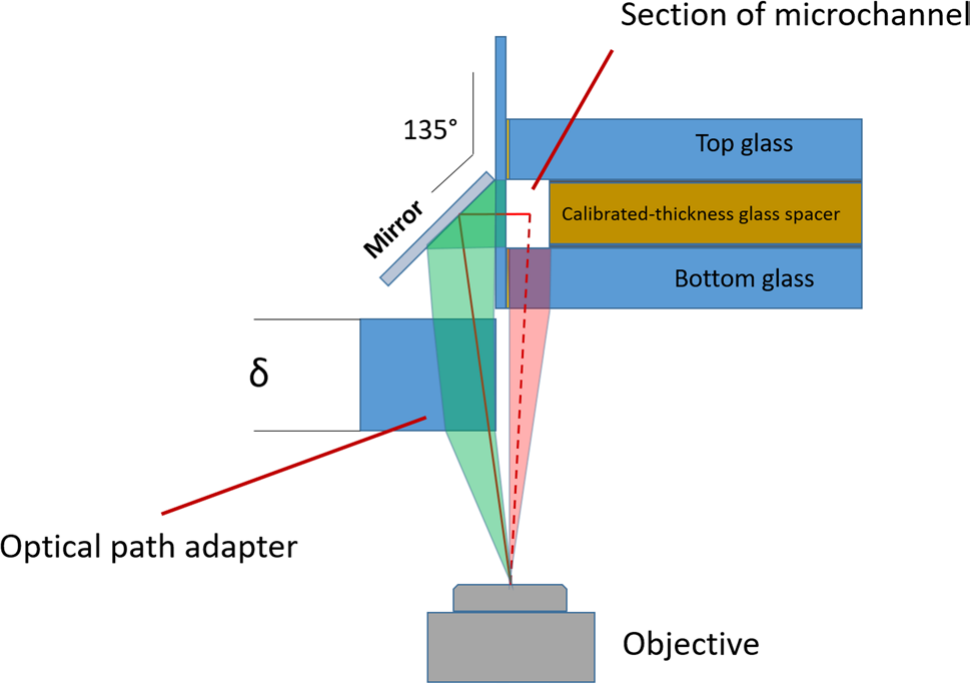
Sketch of the optical system highlighting the optical paths of direct (red dashed line) and reflected (red continuous line) views. The optical path difference is corrected by the optical adapter. (Color figure online)

Microchannels with rectangular cross-sections and different aspect ratio, height and width can be fabricated using calibrated glass spacers of different thickness, highlighted in yellow in sketches of Figs. 2, 3 and 4. Dimensional control of the geometry is achieved using auxiliary calibrated spacers as templates to guarantee channel dimensions and parallelism/orthogonality of the walls. Inlet and outlet sections of the main channel and inlet to the secondary channel are endowed with micro-cannulas for connection through tygon tubes to the respective pumps and waste, tight sealing being ensured by cyanoacrylate glue.

**Fig. 5:**
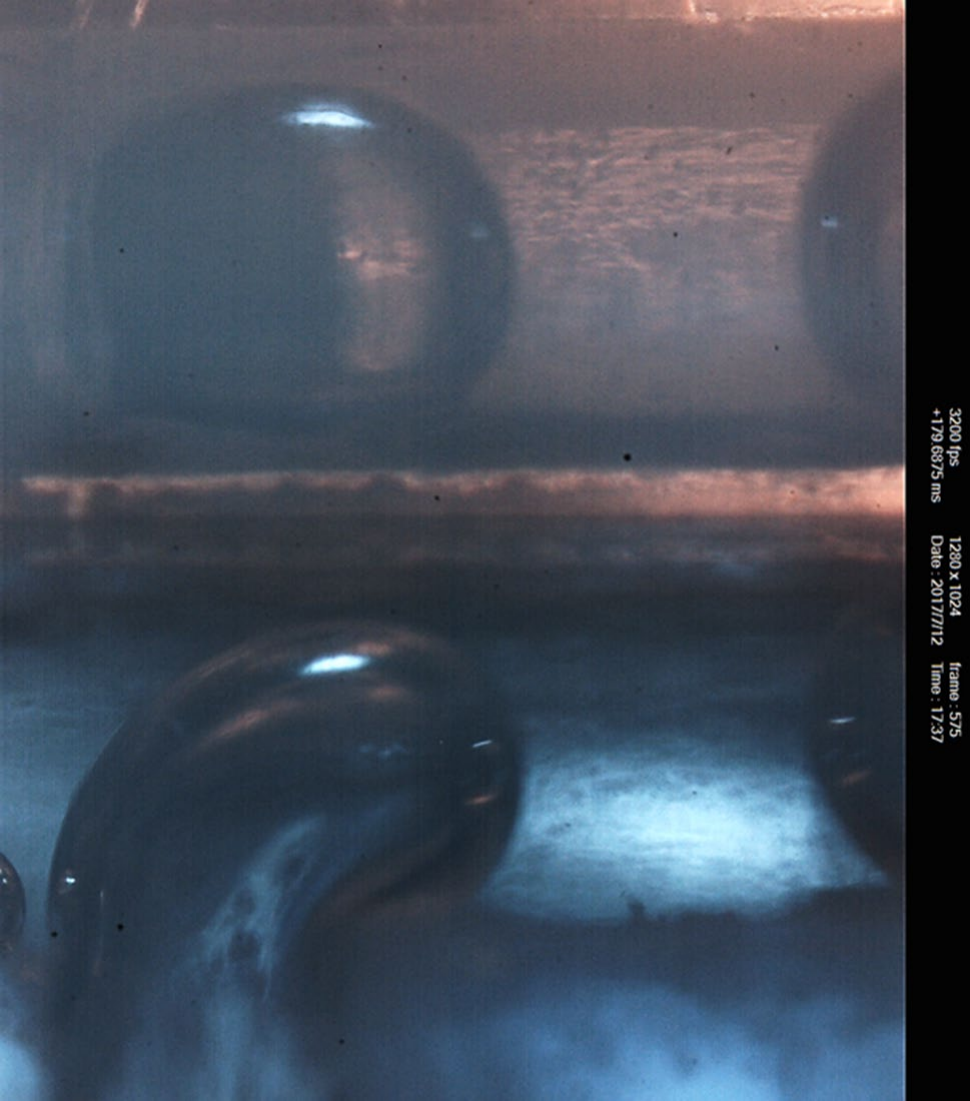
Simultaneous view of the bubble forming at the junction. Raw data correspond to a snapshot taken with the fast camera at $3200\, \mathrm{fps}$. The direct/reflected view is shown in the lower/upper part of the image, respectively. The optical adapter allows both images to be in focus. The different shading is due to the lighting system: microscope lamp in the direct view and auxiliary LED in the reflected view

The double optical access through orthogonal planes allows to visualize the bubble formation and break-up in the two planar views, (*x*–*y*) and (*z*–*x*), respectively, (see the sketch in Fig. 2 for the definition of the coordinate system). The new chip allows for simultaneously capturing both images with a single inverted microscope. This is achieved by integrating a $45^\circ$ inclined mirror on the side slide as shown in Fig. 4. In the present arrangement the (*x*–*y*) view, hereafter called direct view, is directly captured by the objective. The (*z*–*x*) view is instead first reflected on the mirror, reflected view. To simultaneously focus both views, the two optical paths should be identical. This requires the interposition of an optical adapter, here a simple calibrated glass spacer. From the geometry of the optical system, the refraction indices of the materials and the desired positions of the focal planes as well as the thickness of the glass spacer can be easily evaluated, see Appendix A for details.

A good quality micro-mirror is fabricated by evaporating a reflective metal on the surface obtained by cutting a $150\, \upmu \mathrm{m}$-thickness glass slide. A Bulzers evaporator was used to deposit, under vacuum, a 300 Å Aluminium layer on top of a first 150 Å Chrome layer to promote adhesion. In the practical design of the microchip in its basic configuration proposed here, a critical aspect is combining the two conflicting requirements of wide field to include the views in the two orthogonal planes in a single image and sufficient magnification to allow for a precise $\mu$PIV measurement. To this purpose, it is instrumental to minimize the thickness ($150\, \upmu \mathrm{m}$ in the present realization) of the side slide (Fig. 4) that is imaged in the composite view capturing the two orthogonal planes, Fig. 5.

### Flow calibration

**Fig. 6:**
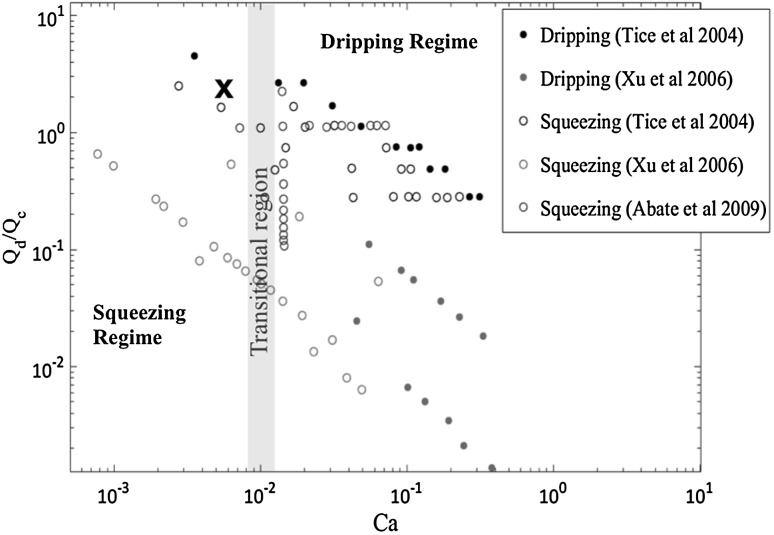
Compilation of literature data corresponding to the different regimes observed in a T-junction (Nunes et al. 2013). The cross indicates the working point used to collect the data in the novel, double view chip

The operational regime of the T-junction depends on the flow rates of the fluids, the viscosity of the two phases, the surface tension and the dimensions of the channels (Garstecki et al. 2006). Figure 6 is a compilation of data (Nunes et al. 2013) showing the phase diagram with the different observed flow regimes where the ordinate is the ratio of disperse, $Q_\mathrm{{d}}$, (gas in our case) to continuous $Q_\mathrm{{c}}$ (liquid) phase flow rates. The abscissa reports the capillary number, $\mathrm{{Ca}} = Q_\mathrm{{c}}\mu /(A \gamma )$, where $\mu$ is the dynamic viscosity of the continuous phase, $\gamma$ the surface tension and $A = h w$ the area of the main channel cross-section. Bubble are formed in two regimes. Below a lower capillary number threshold, the T-junction works in the so-called squeezing regime. Above an upper threshold the bubble starts dripping (dripping regime). Between these two critical values for the capillary number a transitions region, centred around $\mathrm{Ca} \simeq 0.01$, separates the two stable modes of operation. The cross reported in Fig. 6 indicates the conditions used for the data analysis to be discussed in the following, corresponding to $Q_\mathrm{{d}}/Q_\mathrm{{c}} = 1.3$ and capillary number $\mathrm{Ca} = 6.19 \times 10^{-3}$.

To be certain of the operating conditions under which velocity measurements and bubble break-up visualisations are carried out, one should certify the viscosity of the liquid stream and the surface tension at the interface between the two phases that could, in principle, be altered by the fluorescent particles needed for $\mu$PIV. Given the high dilution of the suspension, the viscosity can be estimated by Einstein’s relation $\mu _{\phi }=\mu (1+5/2 \times \phi )$ where $\mu _{\phi }$ is the viscosity of the suspension and $\phi$ is the volume fraction of the suspended particles. As expected, this leads to a negligible change in the viscosity. Surface tension modifications were instead directly quantified exploiting Yurin’s law (Jurin 1718), by measuring the height of the meniscus in a capillary tube partially immersed in the suspension, and were also found to be negligible at the present particle concentration, as expected.

## Results

**Fig. 7:**
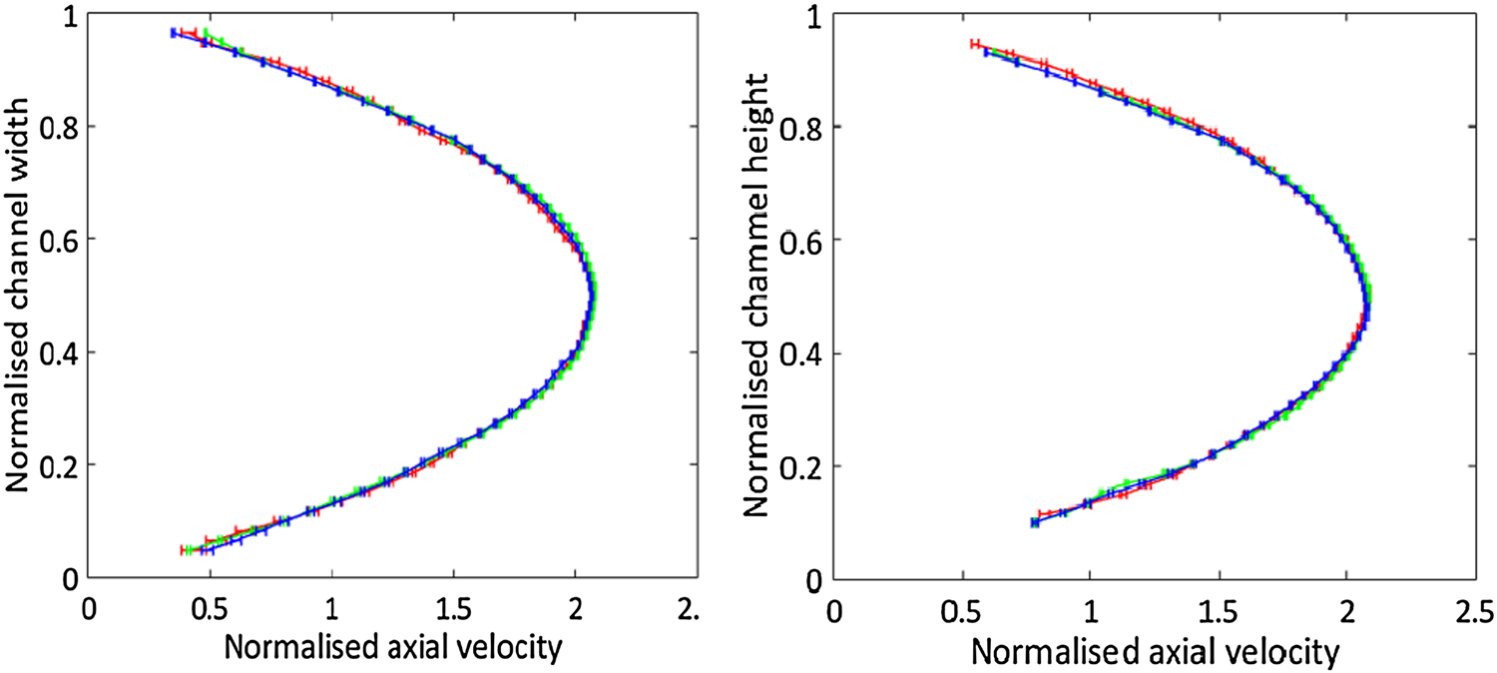
Velocity profiles in the straight channel with square cross-section (length $L = 30 \, \mathrm{mm}$, side $h = w = 800 \, {\upmu \mathrm{{m}}}$). Three different flow rates, $Q = 1,\,2\,\mathrm{{and}}\,3\,\mathrm{ml/min}$, are reported in different colours (red, green and blue, respectively.) In each case, the axial velocity is taken in the middle of the section and is normalised by the corresponding bulk velocity. Left: axial velocity $U_x$ vs *y* at $z = h/2$. The measurement is taken from the direct view. Right: $U_x$ vs *z* at $y = w/2$, measurement taken from the reflected view. The error bars denote ${\bar{\sigma }}/U_{\mathrm{{bulk}}}$, where ${\bar{\sigma }} = {V_x}^{\mathrm{{rms}}}/\sqrt{N_{\mathrm{{sample}}}}$ ($N_{\mathrm{{sample}}} = 450$). (Color figure online)

As a benchmark, results concerning a simple, straight microchannel featuring the same double view with length $L = 30 \, \mathrm{mm}$ and square cross-section with side $w = h = 800\, \upmu \mathrm{m}$ are illustrated first. Ideally, the $\mu$PIV tracer particles should be as small as possible to allow a fast relaxation to the local fluid velocity. In the specific case, assuming a typical flow velocity $U_{\mathrm{{bulk}}} = Q/A = 52\, \mathrm{mm/s}$, the Stokes number of the fluorescent tracers is $St = \rho _\mathrm{{p}} D^2 U_{\mathrm{{bulk}}}/(18 \, \nu h) = 3 \times 10^{-5}$ showing that inertial effects are negligible. Velocity measurements at the microscale may be affected by significant fluctuations due to the Brownian motion of the probing particles. For a colloid of mass $m_\mathrm{{c}}$ the classical theory of Brownian motion predicts the rms (root mean square) fluctuation of a velocity component, say $V_x^{\mathrm{{rms}}}$, to be $V_x^{\mathrm{{rms}}} = \sqrt{k_\mathrm{{B}} T/m_\mathrm{{c}}}$. In the present case, assuming a nominal particle diameter of $4.47 \, \upmu \mathrm{m}$ and a density comparable with water, random fluctuations of intensity $V_x^{\mathrm{{rms}}} = 0.29 \,\mathrm{mm/s}$ are superimposed to the average particle velocity which is the proxy used to estimate the fluid velocity. The particle random motion can be removed from the data by averaging over a collection of samples, under the assumption that the background velocity field is purely deterministic and repeatable, as expected given the low Reynolds number of the flow, in the present case $Re = Q/(h \nu ) = 13$, where *Q* is the flow rate and $\nu$ the kinematic viscosity of the fluid (2-propanol). The maximum number of acquired samples is $N_{\mathrm{{sample}}} = 450$, which gives a confidence interval on the estimated fluid velocity given by ${\bar{\sigma }^{\mathrm{{Brownian}}}_x} = V_x^{\mathrm{{rms}}}/\sqrt{N_{\mathrm{{sample}}}}$. The resulting figure should be compared with the bulk velocity $U_{\mathrm{{bulk}}} = 52 \, \mathrm{mm/s}$ to give ${\bar{\sigma }_x^{\mathrm{{Brownian}}}}/U_{\mathrm{{bulk}}} \simeq 2.6 \times 10^{-4}$. The consequent inaccuracy due to Brownian fluctuations is found one order of magnitude smaller than those due to other error sources to be discussed below.

**Fig. 8:**
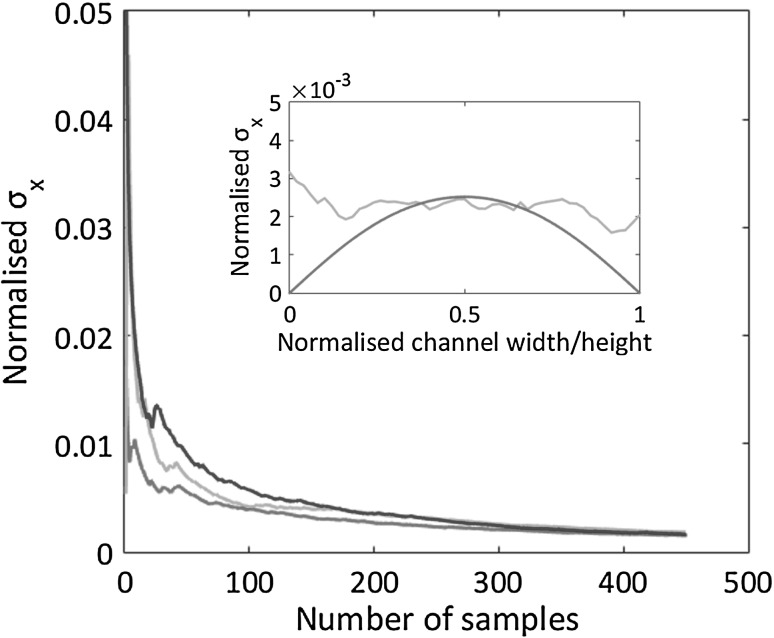
Root mean square axial velocity normalised by the bulk velocity, ${\bar{\sigma }_x}/U_{\mathrm{{bulk}}}$, vs number of samples $N_{\mathrm{{sample}}}$. Colours as in the previous Fig. 7. Data acquired at the midpoint of the channel section $y = w/2, \, z = h/2$. The asymptotic value is ${\bar{\sigma }_x}/U_{\mathrm{{bulk}}} \simeq 2 \times 10^{-3}$. Inset: root mean square axial velocity, experimental data (green curve) and numerical simulation (red). (Color figure online)

The left panel of Fig. 7 shows the axial velocity profiles as obtained from the direct view (*x*–*y*) in the channel symmetry plane, $z = h/2$. The three curves correspond to different flow rates, $Q = 1, \, 2,$ and $3\, \mathrm{ml/min}$ and are normalised by the corresponding bulk velocity. On the right panel, Fig. 7 shows the profiles in the orthogonal view (*x*–*z*, reflected image), now in the symmetry plane $y = w/2$. The profiles, estimated by averaging the velocity over different samples, collapse within the provided confidence interval. For each flow rate, Fig. 8 shows the behaviour of the confidence interval, ${\bar{\sigma }}_x(N_{\mathrm{{sample}}}) = V_x^{\mathrm{{rms}}}/\sqrt{N_{\mathrm{{sample}}}}$, as a function of the number of samples, where $V_x^{\mathrm{{rms}}}$ is the velocity rms obtained from $N_{\mathrm{{sample}}}$ data. The error bars reported on the velocity profiles of Fig. 7 corresponds to $N_{\mathrm{{sample}}} = 450$. The effective number of samples follow from the acquisition of $N_{\mathrm{{frame}}} \simeq 1500$ acquired image couples with occupancy fraction of each interrogation window $f_\mathrm{{o}} = 0.21$. The value of $f_\mathrm{{o}}$ was estimated as explained in Sect. 2 and is confirmed by analysing the raw images from the acquisition system. The inset of the figure shows in green an example of the confidence interval distribution in the transversal direction. The red curve is a theoretical prediction based on a model of the measurement process to be illustrated below.

**Fig. 9:**
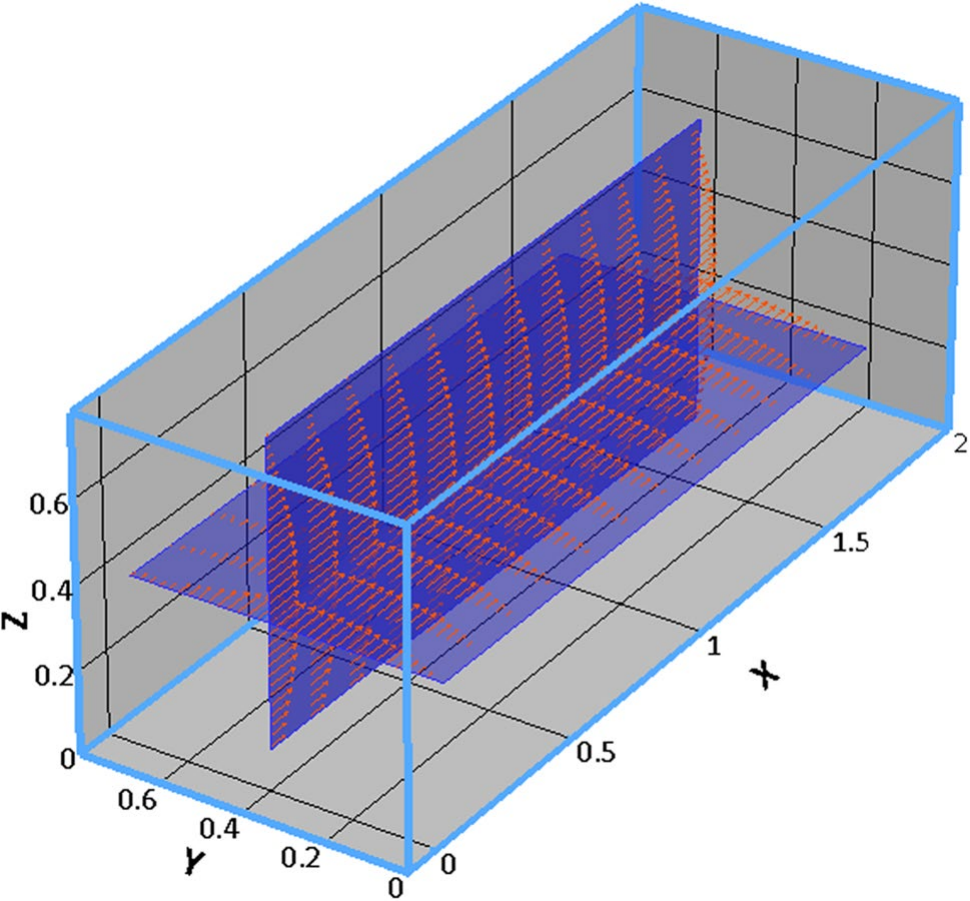
Velocity vectors field reconstruction in the two orthogonal planes corresponding to the direct (*x*–*y*) and reflected (*z*–*x*) views (the measures on axis are in mm). Along the common intersection ($y_0 = w/2, \, z_0 = h/2$) all the three velocity components are available

Figure 9 provides a visual impression of the field showing the two measurement planes and the corresponding in-plane components of the velocity. The two measurement planes intersect along a straight line parallel to the channel axis. The position of the intersection line is determined by maximising the correlation of the axial velocity in the two planes,

$$\begin{aligned} C(y,z) = \frac{\displaystyle \int _0^L U_x^{\mathrm{{direct}}}(x,y) U_x^{\mathrm{{reflected}}}(x,z) \mathrm{{d}}x}{\displaystyle \sqrt{ \int _0^L [U_x^{\mathrm{{direct}}}(x,y) ]^2 \mathrm{{d}}x \int _0^L [U_x^{\mathrm{{reflected}}}(x,z)]^2 \mathrm{{d}}x }}, \end{aligned}$$

where the superscripts direct/reflected denote the plane where the velocity is measured. For the present case of a straight channel, both transversal velocity components, $U_y$ and $U_z$, vanish along the intersection.

The positions of the two orthogonal planes can be independently varied by changing the objective focus and the thickness of the optical path adapter with an accuracy related to the correlation depth. Since, in principle, the coordinates $y_0, \, z_0$ of the intersection line can span the entire channel section, the full three-dimensional field can be, in principle, reconstructed.

**Fig. 10:**
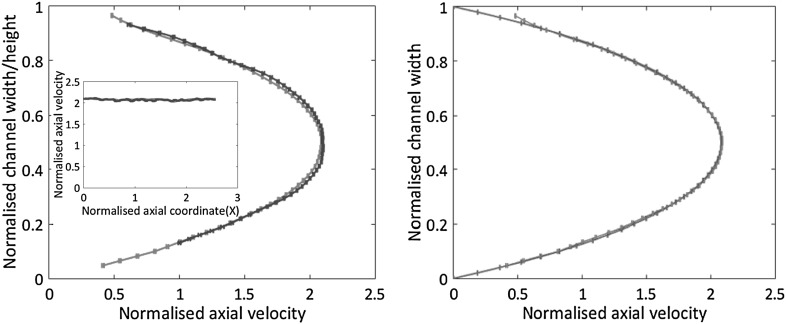
Straight channel with square cross-section, $Q = 2 \,\mathrm{ml/s}$. Left panel: superimposition of the (normalised) axial profiles in the two views (direct and reflected, red and blue, respectively). Error bars defined as in Fig. 7. The inset provides the (normalised) axial velocity along the *x*-line where direct and reflected planes intersect, see Fig. 9. Right panel: comparison of experimental and theoretical profiles (red and cyan, respectively). The theoretical profile is obtained from the analytical solution for the straight square channel, by modelling the measurement procedure and attaching the corresponding theoretical error bars, see text. (Color figure online)

The left panel of Fig. 10 directly compares the velocity profiles across the two orthogonal views [red and blue lines corresponding to direct— $U_x(y)$—and reflected views—$U_x(z)$, respectively]. In a square channel, the velocity profiles on the two orthogonal mid-planes should be identical by symmetry. The comparison confirms that the velocity is correctly reconstructed in both views within the accuracy of the measurement. The inset reports $U_x$ vs the axial coordinate *x* along the intersection line of the two views, illustrating the possibility to simultaneously measure all three velocity components.

**Fig. 11:**
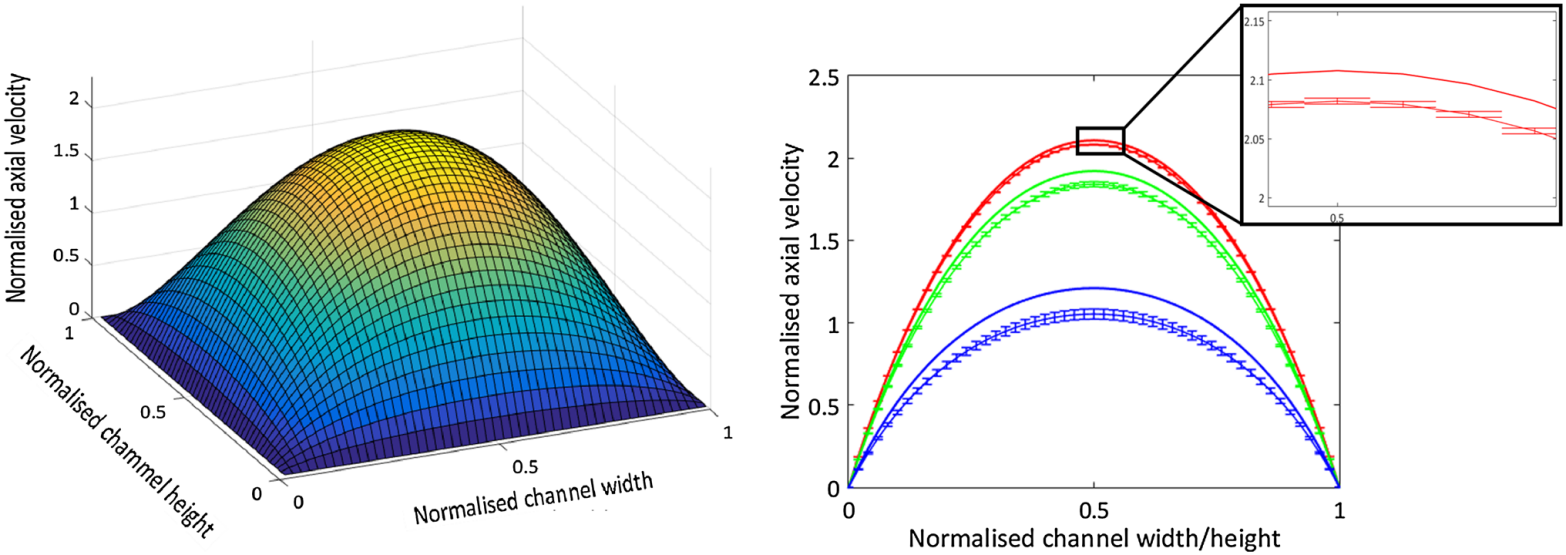
Analytical solution for the Stokes flow in a rectilinear channel with square cross-section. Left panel: axial velocity distribution across the channel section. Right panel: analytical model of the measurement procedure (see text). The measured velocity profiles are reconstructed at $z = 0.25, 0.35$ and $0.5 \, \mathrm{{h}}$. A correlation depth $\ell _\mathrm{{c}} = 0.113\,\mathrm{{h}}$ (see Sect. 2) is assumed and the number of samples is $N_{\mathrm{{sample}}} = 450$. The error bars are computed as illustrated in Appendix C. The exact velocity profile at each *z* position is also shown. Apparently, the statistical error estimated by the error bars is smaller than the systematic error due to averaging the velocity across the finite amplitude slice

The right panel of Fig. 10 provides the comparison between the measured velocity profile and the prediction of a theoretical model of the measurement process. The model is based on the analytical solution for the flow in a straight channel with square cross-section described in details in Appendix B. In summary, the $\mu$PIV signal consists of the fluorescence light emitted by the tracer particles that happen to transit through the region imaged by the optical system. The measurement in, say, the *x*–*y* plane at nominal spanwise location $z_0$ involves the slab $z_0 - \ell _\mathrm{{c}}/2 \le z \le z_0 + \ell _\mathrm{{c}}/2$. Since tracer particles randomly reach the sensitive region (i.e., they are independently and uniformly distributed across the correlation depth), the estimated velocity is in fact the spatial average of the probe velocities across the slab. Apart from Brownian fluctuations, the probe velocity fluctuations are basically a function of the probe position and may be explicitly determined from the analytical velocity and the statistical model reported in Appendix C, see Lima et al. (2006) for a related procedure. Figure 11 illustrates the results of the model. The left panel is the axial velocity distribution across the channel section normalised by the bulk velocity. The right panel shows three profiles at three different spanwise positions. For each case two curves are shown, the (modelled) experimental estimate of the velocity together with the corresponding statistical error bars and the exact velocity at the nominal position. As the model suggests, the main source of error in the conditions of the experiment are due to the averaging of the velocity across the correlation depth. This systematic error component dominates over the statistical error incurred in evaluating the average with a finite sample. As already discussed, the Brownian fluctuation is even smaller, showing that the depth of correlation is the crucial parameter determining the accuracy. As apparent in Fig. 11, the profile taken in the middle of the channel is perfectly captured by the model, see the red curves in the right panel. Both accurately correspond to the actual measurement as shown in the right panel of Fig. 10. Moving towards the side of the channel, the systematic component of the error tends to grow, due to the steeper change of velocity with the position of the probing particle which makes the average across the slab significantly different from the nominal local value.

**Fig. 12:**
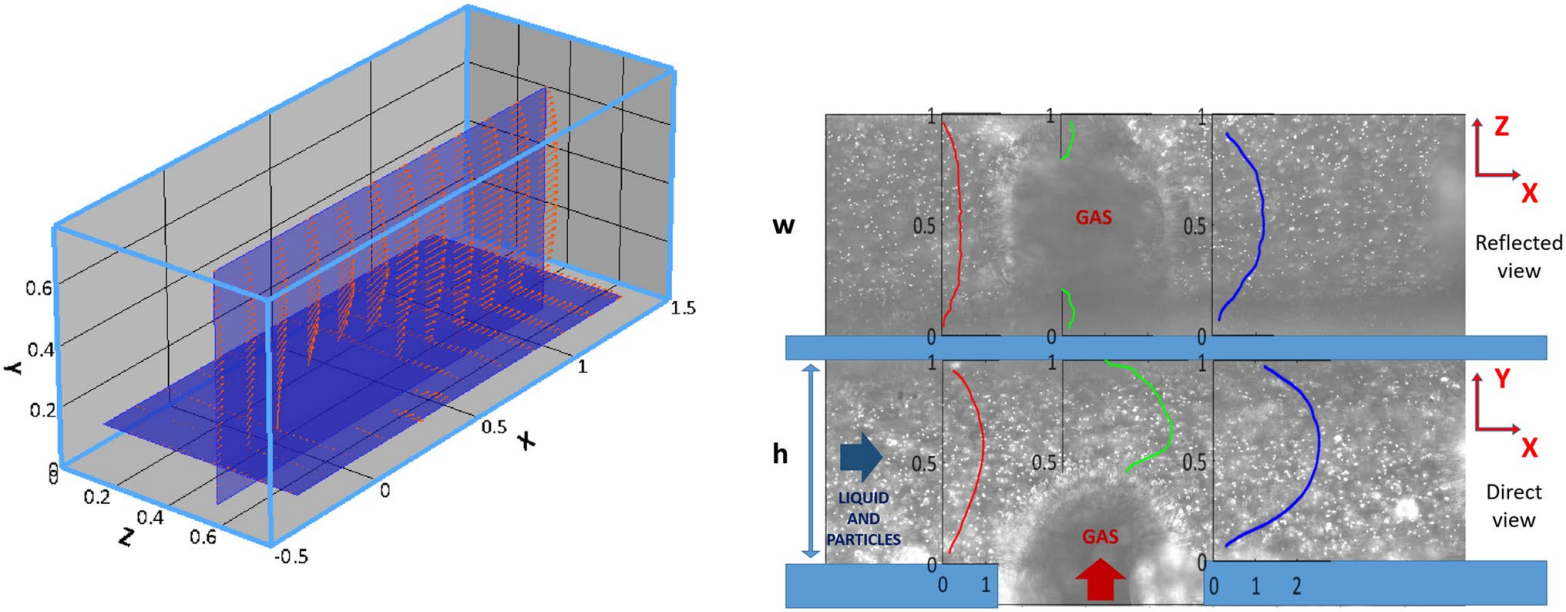
Left panel: tridimensional reconstruction of the measured velocity field in the T-junction (length in mm). The two orthogonal planes are $z_0 = 0.75\,\mathrm{{h}}$ (direct plane) and $y_0 = 0.16 \, w$ (reflected plane). The flow rate of the continuous phase (2-propanol) is $Q_\mathrm{{c}} = 2 \, \mathrm{ml/min}$, the ratio of dispersed to continuous flow rates is $Q_\mathrm{{d}}/Q_\mathrm{{c}} = 1.3$. Average performed over $N_{\mathrm{{sample}}} = 100$ images, in phase with the bubble. The bubble trace can be inferred from the positions of the vectors in the two planes. Right panel: single raw fluorescence image with superimposed velocity profiles in a few sections. Reflected and direct views, top and bottom, respectively. The dots are the fluorescence light scattered from the seeding particles. (Color figure online)

Let us now focus on the T-junction configuration described in Sect. 2. The left panel of Fig. 12 shows the velocity field in the two orthogonal views corresponding to the planes $y_0 = 0.16 \, \mathrm{{w}}$ and $z_0 = 0.75 \, \mathrm{{h}}$. In both planes, the intersection of the plane with the bubble meniscus can be readily figured out. The fields are reconstructed by phase averaging the $\mu$PIV frames corresponding to the same configuration of the bubble. Along the common intersection line, all the three velocity components are measured, providing a time-dependent (depending on the bubble position), three-component reconstruction. As for the case of the straight channel, by changing objective focus and thickness of the optical adapter the entire flow domain can, in principle, be spanned for a complete three-dimensional reconstruction.

At variance with the simpler case of the straight channel, for the T-junction configuration the a priori estimate of the confidence interval is much harder to achieve. The reason is that, due to the geometrically complex and time-dependent flow domain, it is difficult to accurately evaluate the occupancy fraction of the interrogation volume. To circumvent this difficulty, the occupancy fraction was directly evaluated from the raw data. Its typical value turned out to be $f_0 \simeq 0.34$, which yields $N_{\mathrm{{sample}}} \simeq 100$ out of 300 image couples for each bubble configuration. The error bars reported in the velocity profiles to be shown below are based on this information.

**Fig. 13:**
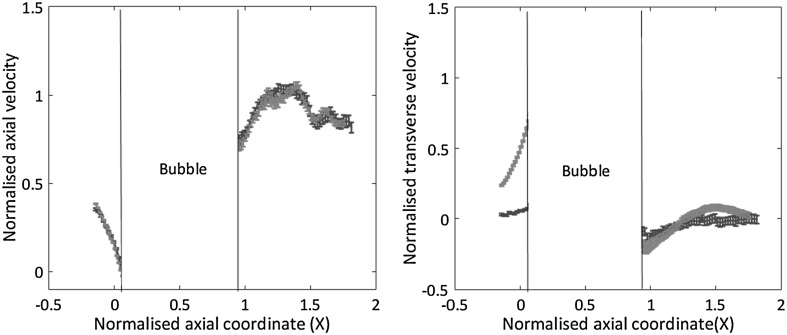
Left panel: axial velocity distribution $U_x$ vs *x* along the intersection line of the two orthogonal planes shown in the left panel of Fig. 12 with related error bars. Direct view, red, and reflected view, blue. Right panel: transversal velocity distribution along the intersection line. The direct view (red) provides $U_y$. The reflected view (blue) provides $U_z$. (Color figure online)

The right panel of Fig. 12 consists of the superposition of a raw image taken from the $\mu$PIV system with three axial velocity profiles at selected stations, one before the bubble (red curve), one on the middle of the bubble (green curve) and the third (blue) behind the bubble. The top/bottom part of the image corresponds to the reflected/direct view, respectively. The axial velocity distribution along the common intersection line is plotted in the left panel of Fig. 13, where the red curve denotes data from the direct (*x*–*y*) view and the blue one corresponds to the reflected (*z*–*x*) view. As for the case of the rectilinear channel, the confidence interval is ${\bar{\sigma }}_x = V_x^{\mathrm{{rms}}}/\sqrt{N_{\mathrm{{sample}}}}$, where $V_x^{\mathrm{{rms}}}$ is the variance of the velocity signal and $N_{\mathrm{{sample}}} \simeq 100$ is the number of samples, related to the total number of acquired frame couples by $N_{\mathrm{{sample}}} = f_0 \, N_{\mathrm{{frame}}}$. The values measured at corresponding positions from the two views are consistent with the expected statistical error, now significantly larger than in the straight channel case due to the reduced number of frames available for a given bubble configuration. The right panel of the figure shows the transverse velocity components along the intersection, with the red line showing $U_y$ from the direct (*x*–*y*) view and the blue line providing $U_z$ from the reflected (*z*–*x*) view.

**Fig. 14:**
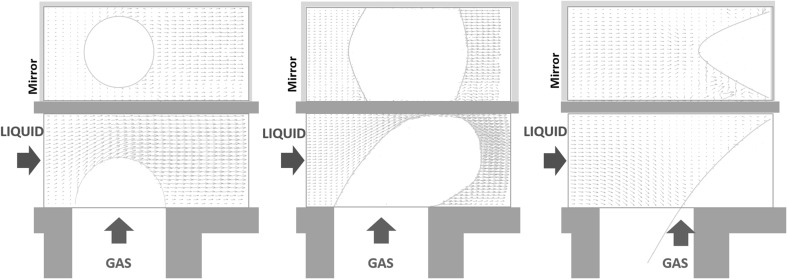
Velocity fields and bubble configurations in the two orthogonal planes at three different phases of the bubble break-up process. The bubble formation frequency is $f = 3\, \mathrm{Hz}$. From left to right $t = 0, \, 4.35, \, 8.7$

Figure 14 provides the spatial distribution of the in-plane velocity vectors together with the trace of the bubble in the two planes. In the present conditions, the bubbles form with frequency $f = 3 \, \mathrm{Hz}$ and the adopted frame acquisition rate is $15 \, \mathrm{Hz}$, corresponding to five images per cycle. In particular, the three plots correspond to dimensionless time instants $t = 0$, $t = 4.35$ and $t=8.7$, with the reference time scale given by $T = h/U_{\mathrm{{bulk}}} = 15.4 \, \mathrm{ms}$.

## Conclusions

An important issue in microfluidics is accessing the three-dimensional structure of the velocity field. For the specific case of a T-junction, the three-dimensional field can be reconstructed using stereo $\mu$PIV as shown in Lindken et al. (2006). Here we provided a proof of principle of a simpler approach which is able to reconstruct the three-dimensional, three-component velocity using a standard $\mu$PIV set-up. A microdevice has been conceived to allow the simultaneous visualisation of the flow in a T-junction along two orthogonal planes. As a specific feature, the novel device allows for capturing both views in a single camera sensor. Half the image corresponds to the direct view, (*x*–*y*) plane. The other half captures the orthogonal view in the (*z*–*x*) plane which is conveyed to the camera objective through an inclined mirror and a suitable optical adapter. The device is used together with a traditional, single camera, $\mu$PIV system able to measure the planar velocity components on the two planes. Beside providing simultaneous views and related velocity components in the two orthogonal planes, all three velocity components are measured along the common intersection line. By adjusting the objective focus and the thickness of the optical adapter, the intersection line can span the three-dimensional flow volume, allowing, in principle, to reconstruct the entire 3D field.

The analysed data were obtained for channels with typical size of $800 \, \upmu \mathrm{m}$ using $4.47 \, \upmu \mathrm{m}$-sized seeding fluorescent particles. In these conditions, the thermally induced Brownian motion of the probes is negligible. The main source of error is found to be related to the $\mu$PIV correlation depth. The adopted fabrication technique allows to reduce the size of the channels down to $100 \, \upmu \mathrm{m}$, due to limit in the assembly precision.

For a $100 \, \upmu \mathrm{m}$ channel, an increased magnification should be used together with smaller tracer particles, e.g., $M = 20 \times$ with $1 \,\upmu \mathrm{m}$ tracer particle diameter. In these conditions, the correlation depth is $\ell _\mathrm{{c}} = 10 \, \upmu \mathrm{m}$, such that $\ell _\mathrm{{c}}/h$ is almost the same as the case of the $800 \, \upmu \mathrm{m}$ channel, explicitly studied here. Assuming the same supply pressure, the expected velocity in the small channel is of the order of $1 \, \mathrm{mm/s}$. On the other hand, the Brownian fluctuations of $1\, \upmu \mathrm{m}$-particles at the same ambient temperature turn out to be $2.7 \, \mathrm{mm/s}$, which is comparable with the fluid velocity. As a consequence, the number of samples needs to be substantially increased to maintain the same statistical accuracy. In any case, like for the larger channel, the main source of error is associated with the correlation depth. In these conditions, a possible strategy to increase the measurement accuracy would be to resort to confocal microscopy (Oishi et al. 2009) which allows for reaching correlation depths of the order of $\ell _\mathrm{{c}} \simeq 5 \, \upmu \mathrm{m}$. One of the interesting aspects of the present device is that it can be easily used in conjunction with confocal $\mu \mathrm{PIV}$, substantially increasing the potentiality of this powerful technique. A second interesting perspective emerges in the opposite limit of a (relatively) large depth of focus in the context of particle tracking. In this case, the two orthogonal views would allow to track the seeding particles in their three-dimensional motion through the measurement volume (Yoon and Kim 2006). A third field of possible application is for benchmarking of other less direct methods to obtain the third velocity component like, e.g., Barnkob et al. (2015), in defocusing $\mu \mathrm{PIV}$. Finally, using image analysis techniques, the present set-up can be used to directly measure the curvature radii of the bubbles, simultaneously with the acquisition of the velocity field.

## Figures and Tables

**Fig. 15:**
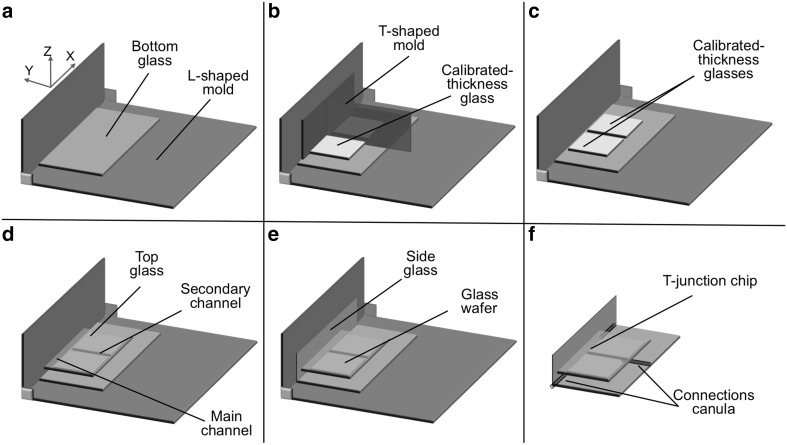
Fabrication procedure. **a** Sketch of the L-shaped template and positioning of the bottom glass. **b** T-shaped mold and positioning of the calibrated-thickness glasses. **c** Main and secondary channels after removal of the T-shaped mold. **d** Positioning of the glass cover. **e** Side glass positioning. **f** Micro-cannulas insertion and overall view of the T-junction device. (Color figure online)

**Fig. 16:**
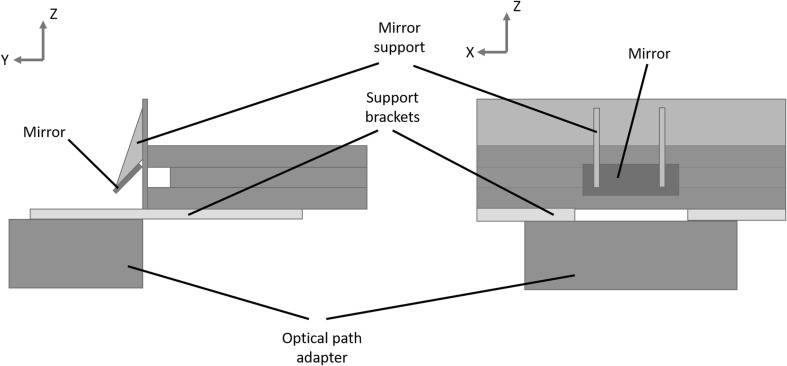
Fabrication procedure. Mirror and optical path adapter positioning

**Fig. 17:**
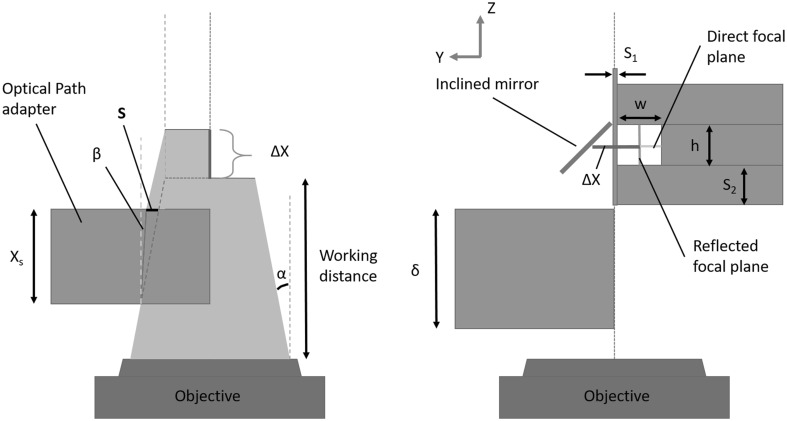
Illustration of the effect of mirror positioning accuracy

## Fabrication procedure

The description of the fabrication procedure is reported step by step here.

- The first step consists in obtaining an L-shaped assembly template (see Fig. 15a). It is built by gluing together two orthogonal glass slides. It is important to achieve a well-cut squaring of the glass slides. Optical microscopy is used to verify the orthogonality of the two walls.
- We place the first glass of the chip in the lower part of the L-shaped mold (bottom glass in Fig. 15a). This slide will therefore form the bottom of our channels. We make sure that the glass is in perfect contact with the orthogonal wall of the L-shaped mold (see Fig. 15a).
- To control the widths of the main and secondary channels of the T-junction, we use a T-shaped mold (in red in Fig.  15b) with two orthogonal slides with the desired size. The T-shaped mold is moved against the vertical wall of the L-shaped template and is pressed on the bottom glass to cancel the play.
- The next step is the positioning of two slides of required thickness (calibrated-thickness glasses in Fig. 15b) on the substrate to define the height of the channels and their position in the *x*–*y* plane. A few micron thickness film of silicon glue is spread over the lower side of the slides by spin coating. The calibrated glasses are moved along the substrate until they get in perfect touch with the T-shaped mold.
- The T-shaped mold is removed (see Fig. 15c) to form main and secondary channel of the T-junction (see Fig. 15d).
- A further slide, glued to the controlled-thickness glasses with silicon glue, is used to cover the channels (top glass in Fig. 15d). The thickness of top and bottom glasses, though not crucial, should be sufficiently thick to allow tight gluing of the lateral side, as explained in the next step. At the end of this step the assembly consists of a glass wafer, made of bottom, top and intermediate calibrated glasses (see Fig. 15e).
- A fifth glass slide is now interposed between the glass wafer and the vertical wall of the L-shaped mold to close the main channel on the *x*–*z* plane (see Fig. 15e). Cyanoacrylate gluing of the side glass is performed in two phases. First, the glue is applied along the contact edge between the top and side glasses. Then, after removing the L-shaped mold, the glue is placed along the lower contact edge. The glue spontaneously spreads on glass, filling the interstices and ensuring tight bonding of the assembly with a thin film.
- Metal micro-cannula are inserted for fluid supply (see Fig. 15f).
- Once the chip is completed, the inclined mirror is introduced on the free side of the Side glass to deflect the *x*–*z* view towards the camera sensor (see Fig. 16). For the present realization a 2 mm $\times$ 20 mm mirror was used to allow imaging of the full width of the channel for a large axial extent. Alignment between mirror and microchannel is verified under optical microscope by spanning along the mirror-slide contact edge.
- The optical path of the reflected view is corrected by interposing a calibrated glass thickness (optical path adapter) between the mirror and the camera sensor (see Fig. 16). The alignment of this adapter is guaranteed by two support brackets. In the paper we analyse two specific cases. The first one concerns the straight channel imaged on the two orthogonal symmetry planes. The second one, concerns the T-junction where both focal planes of direct and reflected views are displaced off the symmetry plane. Each configuration needs a properly tailored glass spacer to compensate for the optical path. The procedure to determine the thickness of the glass spacer is now explained in detail. The working distance of the direct view is given by the objective characteristics, while the working distance of the reflected view has to be properly estimated. The glass spacer with its own refractive index modifies the convergence angle of the light path from the original value $\alpha$ (in absence of the spacer) to the new value $\beta$. The left panel of Fig. 17 shows an idealized configuration. As a consequence of the interposition of the glass spacer a light ray is displaced by *S* parallel to the focal plane, inducing a translation, $\varDelta X$, of the focal plane along the optical axis. *S* follows directly from Snell’s law,
  1 $$\begin{aligned} S=X_\mathrm{{s}} (\tan \alpha - \tan \beta ), \end{aligned}$$
  where $\sin \alpha /\sin \beta =n_2/n_1$ with $n_1$ and $n_2$ the refractive indexes of air and glass respectively. The axial displacement of the focal plane is then expressed as
  2 $$\begin{aligned} \varDelta X = \frac{S}{\tan \alpha }. \end{aligned}$$
  Eqs. (1, 2) can be used to determine the thickness $X_s$ of the glass spacer able to displace the focal plane by a given amount $\varDelta X$, $X_s = \varDelta X \tan (\alpha )/(\tan (\alpha ) - \tan (\beta ))$. In fact, the light ray coming from the reflected view encounters an additional glass layer of thickness $S_1$ (with same refractive index), while the direct view goes through the bottom glass of thickness $S_2$, see right panel of Fig. 17. Taking into account the additional shifts and the change of the propagation medium (air, glass and liquid), the expression for the glass spacer thickness $\delta$ needed to displace the focal plane of the reflected view by $\varDelta X$ relative to the direct view, can be evaluated by equating the light paths of direct view, $l_{\mathrm{{dir}}}$, and reflected view, $l_{\mathrm{{ref}}}$.
  3 $$\begin{aligned} l_{\mathrm{{dir}}}=\, & {} W_\mathrm{{D}} - S_2 \frac{\tan (\alpha ) - \tan (\beta )}{\tan (\alpha )} - \left( \frac{h}{2} + \varDelta z\right) \frac{\tan (\beta ) - \tan (\gamma )}{\tan (\beta )}, \end{aligned}$$
  4 $$\begin{aligned} l_{\mathrm{{ref}}}= \,& {} W_\mathrm{{D}} + W - S_1 \frac{\tan (\alpha ) - \tan (\beta )}{\tan (\alpha )} - \left( \frac{W}{2} + \varDelta y \right) \frac{\tan (\beta ) - \tan (\gamma )}{\tan (\beta )} - \delta \frac{\tan (\alpha ) - \tan (\beta )}{\tan (\alpha )}, \end{aligned}$$
  where $W_\mathrm{{D}}$ is the objective working distance, $\varDelta y$ and $\varDelta z$ are the shifts of the imaged plane with respect to the symmetry planes of the channel and $\sin \beta /\sin \gamma =n_3/n_2$ with $n_3$ the liquid refractive index. Consequently, setting $l_{\mathrm{{dir}}}=l_{\mathrm{{ref}}}$, $\delta$ is given by
  5 $$\begin{aligned} \delta =S_2 - S_1 + W \frac{\tan (\alpha )}{\tan (\alpha ) - \tan (\beta )} + \left( \frac{h}{2} + \varDelta z - \frac{W}{2} - \varDelta y\right) \frac{\tan (\beta ) - \tan (\gamma )}{\tan (\alpha ) - \tan (\beta )}. \end{aligned}$$
  In case the two symmetry planes ($\varDelta y=0$ and $\varDelta z=0$) of the square ($W=h$) channel are imaged, the spacer thickness $\delta _{\mathrm{{sym}}}$ is given by
  6 $$\begin{aligned} \delta _{\mathrm{{sym}}}=S_2 - S_1 + W \frac{\tan (\alpha )}{\tan (\alpha ) - \tan (\beta )}. \end{aligned}$$
  Assuming $n_1=1$, $n_2=1.55$, $n_3=1.36$ (2-prop), $\alpha =0.247 \, \mathrm{rad}$, the glass thickness is $\delta _{\mathrm{{sym}}}=3.3\, \mathrm{{mm}}$. When the focal planes of direct and reflected views are displaced off the symmetry plane by $\varDelta y=-272 \, \upmu \mathrm{m}$ and $\varDelta z=200\, \upmu \mathrm{{m}}$, respectively, the thickness is $\delta =3.15 \, \mathrm{{mm}}$.

## Velocity field in a square duct

The Stokes equations in a rectangular duct ($0 \le y \le w, \, 0 \le z \le h$) infinitely extended in the axial direction *x* result in solving the equation

7 $$\begin{aligned} \mu \nabla ^2_\pi u_x = \frac{\mathrm{{d}}p}{\mathrm{{d}}x}_{|_0}, \end{aligned}$$

with the no-slip boundary conditions

8 $$\begin{aligned} u_x(0,z) = u_x(w,z) = u_x(y,0) = u_x(y,h) = 0. \end{aligned}$$

It easy to show that the cross-flow velocity $\left( u_y,u_z \right)$ is identically zero. In the above equation $\mathrm{{d}}p/\mathrm{{d}}x_{|_0}$ is the constant pressure gradient, $u_x = u_x(y,z)$ is the axial velocity and $\nabla ^2_\pi = \partial ^2/\partial y^2 + \partial ^2/\partial z^2$. The solution of the above problem may be found, e.g., in Bruus (2008). Here we obtain the solution in a slightly different form. We look for the solution in the form $u_x = u_p + u_h$, where a $u_p$ is particular solution satisfying

9 $$\begin{aligned} \mu \nabla ^2_\pi u_\mathrm{{p}} = \frac{\mathrm{{d}}p}{\mathrm{{d}}x}_{|_0}. \end{aligned}$$

A possible choice for $u_\mathrm{{p}}$ is the Hagen–Poiseuille solution for a circular pipe with radius $a = \sqrt{h^2+w^2}/2$, $u_\mathrm{{p}} = - 1/(4 \mu ) \mathrm{{d}}p/\mathrm{{d}}x_{|_0}\left( a^2 - r^2 \right)$, extended to the rectangle of shorter side of length 2*a* circumscribed to the circle. $u_\mathrm{{h}}$ satisfies Laplace equation

10 $$\begin{aligned} \nabla ^2_\pi u_h = 0, \end{aligned}$$

with boundary conditions

11 $$\begin{aligned} \begin{array}{l} u_\mathrm{{h}}(0,z) = -u_\mathrm{{p}}(0,z) = 1/(4 \mu ) \mathrm{{d}}p/\mathrm{{d}}x_{|_0}\left( h z -z^2 \right) \, , y = 0\, , 0 \le z \le h \\ u_\mathrm{{h}}(w,z) = -u_\mathrm{{p}}(w,z) = 1/(4 \mu ) \mathrm{{d}}p/\mathrm{{d}}x_{|_0}\left( h z -z^2 \right) \, , y = w\, , 0 \le z \le h \\ u_\mathrm{{h}}(y,0) = -u_\mathrm{{p}}(y,0) = 1/(4 \mu ) \mathrm{{d}}p/\mathrm{{d}}x_{|_0}\left( w y -y^2 \right) \, ,0 \le y \le w\, , z = 0 \\ u_\mathrm{{h}}(y,h) = -u_\mathrm{{p}}(y,h) = 1/(4 \mu ) \mathrm{{d}}p/\mathrm{{d}}x_{|_0}\left( w y -y^2 \right) \, , 0 \le y \le w\, , z = h. \end{array} \end{aligned}$$

By linearity, the superposition of $u_\mathrm{{p}}$ and $u_\mathrm{{h}}$ satisfies the original differential equation and the corresponding boundary conditions. We may decompose $u_\mathrm{{h}}$ as the sum of four contributions $u_\mathrm{{h}} = u_\mathrm{{t}} + u_\mathrm{{b}} + u_\mathrm{{l}} + u_\mathrm{{r}}$, each of which has assigned values on one side of the rectangle and vanishes on the three remaining ones, e.g.,

$$\begin{aligned}&\nabla ^2_\pi u_t = 0 \\&u_t(0,z) = 0, y = , 0 \le z \le h \\&u_t(w,z) = 0, y = , w \le z \le h \\&u_t(y,0) = 0, 0 \le y \le w, z = 0 \\&u_t(y,h) = 1/(4 \mu ) \mathrm{{d}}p/\mathrm{{d}}x_{|_0} \left( w y -y^2 \right) , 0 \le y \le w\, , z = h. \end{aligned}$$

Exploiting linearity again, the above problem may be solved by superimposing elementary solutions obtained by separation of variables, $u_t = \sum \nolimits _{n=1}^\infty a_{n} \sin (n \pi y/w)\sinh (n \pi z/w)$, which satisfy the differential equation and the homogeneous conditions on three sides ($y=0$, $y=w$, $z=0$) of the boundary. Concerning the fourth side ($z = h$) one has

12 $$\begin{aligned} \sum \limits _{n=1}^\infty a_{n} \sin (n \pi y/w)\sinh (n \pi h/w) = \frac{1}{4 \mu } \frac{\mathrm{{d}}p}{\mathrm{{d}}x_{|_0}} \left( w y -y^2 \right) , \end{aligned}$$

which yields the coefficients

13 $$\begin{aligned} a_{n}= & {} \frac{1}{4 \mu \sinh (n \pi h/w)} \frac{\mathrm{{d}}p}{\mathrm{{d}}x_{|_0}} \frac{2}{w} \int _0^w \left( w y -y^2 \right) \sin (n \pi y/w) dy \nonumber \\= & {} \frac{1}{4 \mu \sinh (n \pi h/w)} \frac{\mathrm{{d}}p}{\mathrm{{d}}x_{|_0}} \frac{4 w^2}{\pi ^3} \left[ 1 - (-1)^n \right] . \end{aligned}$$

The other solutions are easily obtained by symmetry. In particular, the elementary solution with non-vanishing data at the bottom side, $u_\mathrm{{b}}$, is obtained from $u_t$ by the substitution $z\rightarrow h -z$, $u_\mathrm{{b}} = \sum \nolimits _{n=1}^\infty a_{n} \sin (n \pi y/w)\sinh [n \pi (h/w - z/w)]$ with the same coefficients $a_n$ as before. The solution with non-vanishing data on the right side of the rectangle is instead obtained from $u_t$ with the substitutions $z \rightarrow y$, $y \rightarrow z$, $w \rightarrow h$, $h \rightarrow w$, $u_r = \sum \nolimits _{n=1}^\infty b_{n} \sin (n \pi z/h)\sinh (n \pi y/h)$ with coefficients given by

14 $$\begin{aligned} b_{n} = \frac{1}{4 \mu \sinh (n \pi w/h)} \frac{\mathrm{{d}}p}{\mathrm{{d}}x_{|_0}} \frac{4 h^2}{\pi ^3} \left[ 1 - (-1)^n \right] \ . \end{aligned}$$

Finally $u_\mathrm{{l}}$ follows as $u_\mathrm{{l}} = \sum \nolimits _{n=1}^\infty b_{n} \sin (n \pi z/h)\sinh [n \pi (w/h - y/h)]$, with same $b_n$ coefficients. Eventually, the solution of the original problem (7, 8) shown in the left panel of Fig. 11 is

15 $$\begin{aligned} \frac{u_x(y,z)}{- 1/\mu \mathrm{{d}}p/\mathrm{{d}}x_{|_0}}= & {} \frac{w y-y^2 + hz-z^2}{4} \qquad \qquad \qquad \qquad \nonumber \\&- w^2 \sum \limits _{n=1}^\infty \frac{1 - (-1)^n}{\pi ^3} \sin (n \pi y/w)\frac{\sinh (n \pi z/w) + \sinh \left[ n \pi \left( h/w - z/w\right) \right] }{\sinh (n \pi h/w) } \nonumber \\&- h^2 \sum \limits _{n=1}^\infty \frac{1 - (-1)^n}{\pi ^3} \sin (n \pi z/h)\frac{\sinh (n \pi y/h) + \sinh 
\left[ n \pi \left( w/h - y/h\right) \right] }{\sinh (n \pi w/h) }, \end{aligned}$$

with flow rate $Q = \int _0^w \int _0^h u_x(y,z) \mathrm{{d}}y \mathrm{{d}}z$ given by

16 $$\begin{aligned} \frac{Q}{\displaystyle - \frac{1}{\mu }\frac{\mathrm{{d}}p}{\mathrm{{d}}x}_{|_0} \left( h w\right) ^2}= & {} \frac{1}{24} \left( \frac{h}{w} + \frac{w}{h} \right) \qquad \qquad \qquad \qquad \nonumber \\&-\frac{8}{\pi ^5} \sum \limits _{n=0}^\infty \left\{ \left( \frac{w}{h}\right) ^2 \frac{\cosh \left[ (2n+1) \pi \frac{w}{h}\right] -1}{\sinh \left[ (2n+1) \pi \frac{w}{h}\right] } + \left( \frac{h}{w}\right) ^2 \frac{\cosh \left[ (2n+1) \pi \frac{h}{w}\right] -1}{\sinh \left[ (2n+1) \pi \frac{h}{w}\right] } \right\} \ . \end{aligned}$$

## Modelling the $\mu$PIV statistics

The finite correlation depth $\ell _\mathrm{{c}}$ of the $\mu$PIV, estimated on the order of $90 \, \upmu \mathrm{m}$ in Sect. 2, induces noise in the measurements since all the tracer particles in a slice of thickness $\ell _\mathrm{{c}}$ contribute to the measured velocity on a given nominal plane. Focusing on the *z*–*x* plane for the sake of definiteness, the measured streamwise velocity at position $y_0$ will be the average of signals coming from all the particles with position $y_0-\ell _\mathrm{{c}}/2 \le y \le y_0 + \ell _\mathrm{{c}}/2$. A reasonably good model is to assume that the probability to find the particles in the slice is uniform,

17 $$\begin{aligned} p(y) = \left\{ \begin{array}{lll} \frac{1}{\displaystyle \ell _\mathrm{{c}}} &{} \mathrm{for} &{} y_0-\ell _\mathrm{{c}}/2 \le y \le y_0 + \ell _\mathrm{{c}}/2 \\ 0 &{} \mathrm{otherwise} &{} \end{array} \right. . \end{aligned}$$

Since each tracer particle is carried along with the local fluid velocity, $u_x(y,z)$, the probability distribution function (pdf) of the particle velocity at position *z* that contribute to the acquired signal at the nominal position $y_0$ is

18 $$\begin{aligned} p(v_x,y_0,z) = \int \delta \left[ v_x - u_x(y,z) \right] p(y) \mathrm{{d}}y \ . \end{aligned}$$

The average velocity of the particles in the slice centred at $y_0$ is then

19 $$\begin{aligned} \langle v_x \rangle (y_0)& = {} \int v_x p(v_x) \mathrm{{d}}v_x = \int \int v_x \delta \left[ v_x - u_x(y,z) \right] p(y) \mathrm{{d}}y \mathrm{{d}}v_x \nonumber \\ & = {} \int u_x(y,z) p(y) \mathrm{{d}}y = \frac{1}{\ell _\mathrm{{c}}} \int _{-\ell _\mathrm{{c}}/2}^{\ell _\mathrm{{c}}/2} u_x(y,z) \mathrm{{d}}y. \end{aligned}$$

Analogously, the particle velocity variance is

20 $$\begin{aligned} \langle {v'}_x^2 \rangle (y_0)=\, &{} \int {v'}_x^2 p(v_x) \mathrm{{d}}v_x = \int \left( v_x - \langle v_x\rangle \right) ^2 p(v_x) \mathrm{{d}}v_x\nonumber \\ \int \left[ u_x(y,z) - \langle v_x\rangle \right] ^2 p(y) \mathrm{{d}}y= \,& {} \frac{1}{\ell _c} \int _{-\ell _\mathrm{{c}}/2}^{\ell _\mathrm{{c}}/2} \left[ u_x(y,z) - \langle v_x\rangle \right] ^2 \mathrm{{d}}y. \end{aligned}$$

The estimator of the fluid velocity at the nominal position $(y_0,z)$ corresponds to the arithmetic average of the velocity $v_x^{(k)}\, , \; k=1, \ldots ,N$, of *N* tracer particles as measured by $\mu$PIV,

21 $$\begin{aligned} {\bar{u}}_x(y_0,z) = \frac{1}{N} \sum \limits _{k=1}^N v_x^{(k)}. \end{aligned}$$

Under the assumption of independently distributed particle positions, from the central limit theorem the estimator is seen to fluctuate with variance

22 $$\begin{aligned} {\bar{\sigma }}_x(y_0,z) = \sqrt{\langle {{\bar{u}}_x(y_0,z)'}^2 \rangle } = \frac{\sigma _x(y_0,z)}{\sqrt{N}}, \end{aligned}$$

where $\sigma _x = \sqrt{\langle {v'_x}^2\rangle }$. The analytic solution reported in Sect. C for the square duct allows to obtain both the mean value $\langle v_x\rangle$, i.e.,

23 $$\begin{aligned} \langle {\bar{u}}_x(y_0,z)\rangle = \langle v_x\rangle \, , \end{aligned}$$

and the variance $\langle {v'_x}^2\rangle$, i.e., the statistical error bar $e = 3 {\bar{\sigma }}_x(y_0,z)$ on the estimated velocity ${\bar{u}}_(y_0,z)$. Moreover the estimate with *N* samples can be compared with the true solution $u_x(y_0,z)$. The results of the right panel of Fig. 11 show that, with increasing the number of samples, the estimated velocity statistically converges toward the average across the slice. The statistical fluctuation becomes negligible and the error in reconstructing the velocity is dominated by the systematic difference between the local fluid velocity and its average across the finite thickness slice.
